# OXA-48–Producing Uropathogenic *Escherichia coli* Sequence Type 127, the Netherlands, 2015–2022

**DOI:** 10.3201/eid2912.231114

**Published:** 2023-12

**Authors:** Marlies Mulder, Daan W. Notermans, Cornelia C.H. Wielders, Jeroen Bos, Sandra Witteveen, Varisha A. Ganesh, Fabian Landman, Angela de Haan, Caroline Schneeberger-van der Linden, Antoni P.A. Hendrickx, on behalf of the Dutch CPE Surveillance Study Group

**Affiliations:** Maastricht University Medical Center+, Maastricht, the Netherlands (M. Mulder);; National Institute for Public Health and the Environment (RIVM), Bilthoven, the Netherlands (M. Mulder, D.W. Notermans, C.C.H. Wielders, J. Bos, S. Witteveen, V.A. Ganesh, F. Landman, A. de Haan, C. Schneeberger-van der Linden, A.P.A. Hendrickx)

**Keywords:** *Escherichia coli*, uropathogenic *Escherichia coli*, UPEC, carbapenemase-producing Enterobacterales, OXA-48, ST127, bacteria, antimicrobial resistance, the Netherlands

## Abstract

During 2015–2022, a genetic cluster of OXA-48–producing uropathogenic *Escherichia coli* sequence type 127 spread throughout the Netherlands. The 20 isolates we investigated originated mainly from urine, belonged to Clermont phylotype B2, and carried 18 genes encoding putative uropathogenicity factors. The isolates were susceptible to first-choice antimicrobial drugs for urinary tract infections.

We recently described OXA-244 carbapenemase-producing *Escherichia coli* sequence type (ST) 38 with putative uropathogenicity factors (*1*). Here we report a genetic cluster of 20 OXA-48–producing uropathogenic *Escherichia coli* (UPEC) ST127 isolates in the Netherlands.

Medical microbiology laboratories in the Netherlands are requested to submit isolates with suspected carbapenemase production to the National Institute for Public Health and the Environment (RIVM) as part of the carbapenemase-producing *Enterobacterales* (CPE). For all isolates, we perform meropenem Etest, carbapenem inactivation method, next-generation sequencing (NGS; lllumina, https://www.illumina.com), and long-read sequencing (Oxford Nanopore Technologies, https://www.nanoporetech.com). We use NGS data to analyze the Clermont phylotype (*2*), core-genome single-nucleotide polymorphisms, classical multilocus sequence typing (MLST) STs, and in-house *E. coli* whole-genome MLST (wgMLST) types (*1*,*3*). We also evaluated presence of antimicrobial resistance genes (AMRfinder, https://www.ncbi.nlm.nih.gov/pathogens/antimicrobial-resistance/AMRFinder), plasmid replicons (PlasmidFinder, https://cge.food.dtu.dk/services/PlasmidFinder), and 31 previously described putative uropathogenicity factors (PUFs) using an in-house PUFfinder (*4*). For identity/query >90%, we scored the PUF gene as present. 

During January 1, 2015–December 31, 2022, we sequenced 799 carbapenemase-producing *E. coli* by using NGS; 258 (32%) carried a *bla*_OXA-48_ gene, of which 24 were ST127. According to wgMLST, 20 of the *bla*_OXA-48_–carrying ST127 isolates formed a genetic cluster (Appendix 1 Figure, panel A) and were sent to the RIVM during October 2015–December 2022 (Appendix 1 Figure, panel B). Allelic distance in the cluster was 3–20, and isolates differed by 3–46 core-genome single-nucleotide polymorphisms (Appendix 2 Table 2). When we compared the 20 cluster isolates with 603 international *E. coli* ST127 isolates (Enterobase, https://enterobase.warwick.ac.uk), they clustered with 3 isolates: Ireland (2016), United States (2019), and Spain (2019) (Appendix 2 Table 3). All were sensitive to meropenem (European Committee on Antimicrobial Susceptibility Testing, https://www.eucast.org); MICs were 0.125–0.38 mg/L (*5*). All grew on OXA-48 agar but not carbapenemase agar (CHROMID OXA-48/CHROMID CARBA; bioMérieux, https://www.biomerieux.com) and produced carbapenemase according to the carbapenem inactivation method. Nanopore sequencing yielded 10/20 circular assemblies, which revealed a chromosomal copy of the *mdf(A)-* and the *bla*_OXA-48_ genes. The *bla*_OXA-48_ gene is flanked by IS1/tnp-IS1B and inserted in a variable ≈148-kb region of the chromosome (Appendix 1 Figure, panel C; Appendix 2 Tables 1, 4). Of the 20 isolates, 18 lacked plasmid replicons.

The median age of the 11 male and 9 female patients was 57 (range 3–87) years; patients lived throughout the Netherlands (Appendix 1 Figure, panel D). Cultures were submitted by general practitioners (8/20) and hospitals (12/20). Two patients were recently hospitalized in Morocco; no travel history was reported for the other patients, although 1 was born in Morocco and 1 in Turkey. 

Most isolates were from urine (12/20), followed by perineal/rectal swab samples (4/20), blood (3/20), and wounds (1/20). Of the 20 cultures, 12 were diagnostic, 5 were screening, and 3 were for unknown purpose. Two patients had recurrent urinary tract infections (UTIs). All isolates were type O6:H31 and Clermont phylotype B2, the most common Clermont phylotype associated with UPEC in the United States and Europe (*4*,*6*). A variety of PUFs, associated with UPEC, were detected in cluster isolates (Appendix 2 Table 5, Appendix 1 Figure, panel E), including adhesins (e.g., *sfaH*, pili *papGII*/*papGIII*), toxins (e.g., α-hemolysin, cytotoxic necrotizing factor-1, and *E. coli* uropathogenic-specific protein) (*4*,*7*,*8*). Cluster isolates carried significantly more (mean 18) PUFs, than the other *E. coli* isolates from CPE surveillance (mean nonurine isolates, 7; urine isolates, 9; previously reported OXA-244 *E. coli* ST38 isolates, 8; p<0.001 by Mann-Whitney U-test) (Appendix 1 Figure, panel F) (*1*). We identified additional uropathogenicity determinants curli, type-I fimbriae, S-fimbriae, flagella, and group 2 capsule genes but not group 3 capsule genes. Eighteen isolates phenotypically produced hemolysin, visible as β-hemolysis on blood agar (Appendix 1 Figure, panel F), in line with in silico genetic analyses (Appendix 1 Figure, panel E).

Antimicrobial susceptibility pattern was known for 13 isolates in the Infectious Diseases Surveillance Information System–Antimicrobial Resistance in the Netherlands (https://www.rivm.nl/isis-ar). All were phenotypically resistant to penicillins/penicillin combinations (e.g., amoxicillin/clavulanic acid and piperacillin/tazobactam) but susceptible to oral first-choice antimicrobial drugs for UTIs in the Netherlands (e.g., nitrofurantoin, fosfomycin, ciprofloxacin, sulfamethoxazole/trimethoprim) (Appendix 1 Figure, panel E). Prevalence of UPEC in the Netherlands is most likely underestimated because general practitioners in the Netherlands usually send cultures only when treatment with first-choice drugs fails. Although UTIs are not known to be contagious, *E. coli* can spread and cause UTI outbreaks (caused by a specific *E. coli* strain in several communities), for which an association with food has been suggested (*9*). A New Zealand study described an outbreak in which MLST identified 77 multidrug-resistant *E. coli* isolates (*10*).

We demonstrated ongoing dissemination of OXA-48–producing and hemolysin-producing UPEC ST127 from Clermont phylotype B2 with 18/31 PUFs in patients across the Netherlands with no direct epidemiologic link. The origin of the cluster is unknown, but international spread is possible. Low-level resistance and growth only on OXA-48 agar suggests that this carbapenemase-producing UPEC may be missed and the actual size of this cluster may be underestimated.

Appendix 1 Additional information for study of OXA-48–producing uropathogenic *Escherichia coli* sequence type 127, the Netherlands, 2015–2022.

Appendix 2 Sequencing and related data for study of OXA-48–producing uropathogenic *Escherichia coli* sequence type 127, the Netherlands, 2015–2022.
