# Supplementary material for: OXA-48–Producing Uropathogenic Escherichia coli Sequence Type 127, the Netherlands, 2015–2022
Source: Emerg Infect Dis. 2023 Dec;29(12):2563–5. doi: 10.3201/eid2912.231114 (PMC10683799; doi:10.3201/eid2912.231114)
Supplement: Appendix 1 — Additional information for study of OXA-48–producing uropathogenic Escherichia coli sequence type 127, the Netherlands, 2015–2022. [file 23-1114-Techapp-s1.pdf]

Article DOI: https://doi.org/10.3201/eid2912.231114

EID cannot ensure accessibility for supplementary materials supplied by authors. Readers who have difficulty accessing supplementary content should contact the authors for assistance.

OXA-48–Producing Uropathogenic *Escherichia coli* Sequence Type 127, the Netherlands, 2015–2022

Appendix 1

Dutch CPE Surveillance Study Group

- W. van den Bijllaardt, Amphia Hospital, Microvida Laboratory for Microbiology, Breda
- A.L.E. van Arkel, ADRZ medisch centrum, Department of Medical Microbiology, Goes
- M.A. Leversteijn-van Hall, Alrijne Hospital, Department of Medical Microbiology, Leiden
- R. van Mansfeld, Amsterdam UMC - location AMC, Department of Medical Microbiology and Infection Control, Amsterdam
- K. van Dijk, Amsterdam UMC - location Vumc, Department of Medical Microbiology and Infection Control, Amsterdam
- B. Zwart, Atalmedial, Department of Medical Microbiology, Amsterdam
- B.M.W. Diederen, Bravis Hospital/ZorgSaam Hospital Zeeuws-Vlaanderen, Department of Medical Microbiology, Roosendaal/Terneuzen
- J.W. Dorigo-Zetsma, TergooiMC, Central Bacteriology and Serology Laboratory, Hilversum
- D.W. Notermans, Centre for Infectious Disease Control, National Institute for Public Health and the Environment, Bilthoven

- A. Ott, Certe, Medical Microbiology Groningen, Drenthe, Groningen
- W. Ang, Comicro, Department of Medical Microbiology, Hoorn
- J. da Silva, Deventer Hospital, Department of Medical Microbiology, Deventer
- A.L.M. Vlek, Diakonessenhuis Utrecht, Department of Medical Microbiology and Immunology, Utrecht
- A.G.M. Buiting, Elisabeth-TweeSteden (ETZ) Hospital, Department of Medical Microbiology and Immunology, Tilburg
- L.G.M. Bode, Erasmus University Medical Center, Department of Medical Microbiology and Infectious Diseases, Rotterdam
- S. Paltansing, Franciscus Gasthuis & Vlietland, Department of Medical Microbiology and Infection Control, Rotterdam
- A.J. van Griethuysen, Gelderse Vallei Hospital, Department of Medical Microbiology, Ede
- M. den Reijer, Star-shl diagnostic center, Department of Medical Microbiology, Rotterdam
- M.J.C.A. van Trijp, Groene Hart Hospital, Department of Medical Microbiology and Infection Prevention, Gouda
- M. Wong, Haga Hospital, Department of Medical Microbiology, 's-Gravenhage
- A.E. Muller, HMC Westeinde Hospital, Department of Medical Microbiology, 's-Gravenhage
- M.P.M. van der Linden, IJsselland hospital, Department of Medical Microbiology, Capelle a/d IJssel
- M. van Rijn, Ikazia Hospital, Department of Medical Microbiology, Rotterdam
- S.B. Debast, Isala Hospital, Laboratory of Medical Microbiology and Infectious Diseases, Zwolle
- K. Waar, Certe, Medical Microbiology Friesland | Noordoostpolder, Leeuwarden

- E. Kolwijck, Jeroen Bosch Hospital, Department of Medical Microbiology and Infection Control, 's-Hertogenbosch
- N. Al Naiemi, LabMicTA, Regional Laboratory of Microbiology Twente Achterhoek, Hengelo
- T. Schulin, Laurentius Hospital, Department of Medical Microbiology, Roermond
- S. Dinant, Maasstad Hospital, Department of Medical Microbiology, Rotterdam
- S.P. van Mens, Maastricht University Medical Centre, Department of Medical Microbiology, Infectious Diseases & Infection Prevention, Maastricht
- DC Melles, Meander Medical Center, Department of Medical Microbiology, Amersfoort
- M.P.A. van Meer, Rijnstate Hospital, Laboratory for Medical Microbiology and Immunology, Velp
- J.W.T. Cohen Stuart, Noordwest Ziekenhuisgroep, Department of Medical Microbiology, Alkmaar
- P. Gruteke, OLVG Lab BV, Department of Medical Microbiology, Amsterdam
- A. Jansz, Eurofins PAMM, Department of Medical Microbiology, Veldhoven
- A. van Dam, Public Health Service, Public Health Laboratory, Amsterdam
- I. Maat, Radboud University Medical Center, Department of Medical Microbiology, Nijmegen
- B. Maraha, Albert Schweitzer Hospital, Department of Medical Microbiology, Dordrecht
- J.R. Lo Ten Foe, Gelre Hospital, Department of Medical Microbiology and Infection Control, Apeldoorn
- J.C. Sinnige, Regional Laboratory of Public Health, Department of Medical Microbiology, Haarlem
- E. van der Vorm, Reinier de Graaf Groep, Department of Medical Microbiology, Delft

- M. de Graaf, Saltro Diagnostic Centre, Department of Medical Microbiology, Utrecht
- E. de Jong, Slingeland Hospital, Department of Medical Microbiology, Doetinchem
- S.J. Vainio, St Antonius Hospital, Department of Medical Microbiology and Immunology, Nieuwegein
- E. Heikens, St Jansdal Hospital, Department of Medical Microbiology, Harderwijk
- R. Steingrover, St. Maarten Laboratory Services, Department of Medical Microbiology, Cay Hill (St. Maarten)
- A. Troelstra, University Medical Center Utrecht, Department of Medical Microbiology, Utrecht
- E. Bathoorn, University of Groningen, Department of Medical Microbiology, Groningen
- J. de Vries, VieCuri Medical Center, Department of Medical Microbiology, Venlo
- D.W. van Dam, Zuyderland Medical Centre, Department of Medical Microbiology and Infection Control, Sittard-Geleen
- E.I.G.B. de Brauwer, Zuyderland Medical Centre, Department of Medical Microbiology and Infection Control, Heerlen
- NN, Analytical Diagnostic Center N.V. Curaçao, Department of Medical Microbiology, Willemstad (Curaçao)
- H. Berkhout, Canisius Wilhelmina Hospital, Department of Medical Microbiology and Infectious Diseases, Nijmegen

References

1. Notermans DW, Schoffelen AF, Landman F, Wielders CCH, Witteveen S, Ganesh VA, et al.; Dutch CPE Surveillance Study Group. A genetic cluster of OXA-244 carbapenemase-producing *Escherichia coli* ST38 with putative uropathogenicity factors in the Netherlands. J Antimicrob Chemother. 2022;77:3205–8. PubMed https://doi.org/10.1093/jac/dkac307

2. Schreiber HL IV, Conover MS, Chou WC, Hibbing ME, Manson AL, Dodson KW, et al. Bacterial virulence phenotypes of *Escherichia coli* and host susceptibility determine risk for urinary tract infections. Sci Transl Med. 2017;9:eaaf1283. PubMed https://doi.org/10.1126/scitranslmed.aaf1283

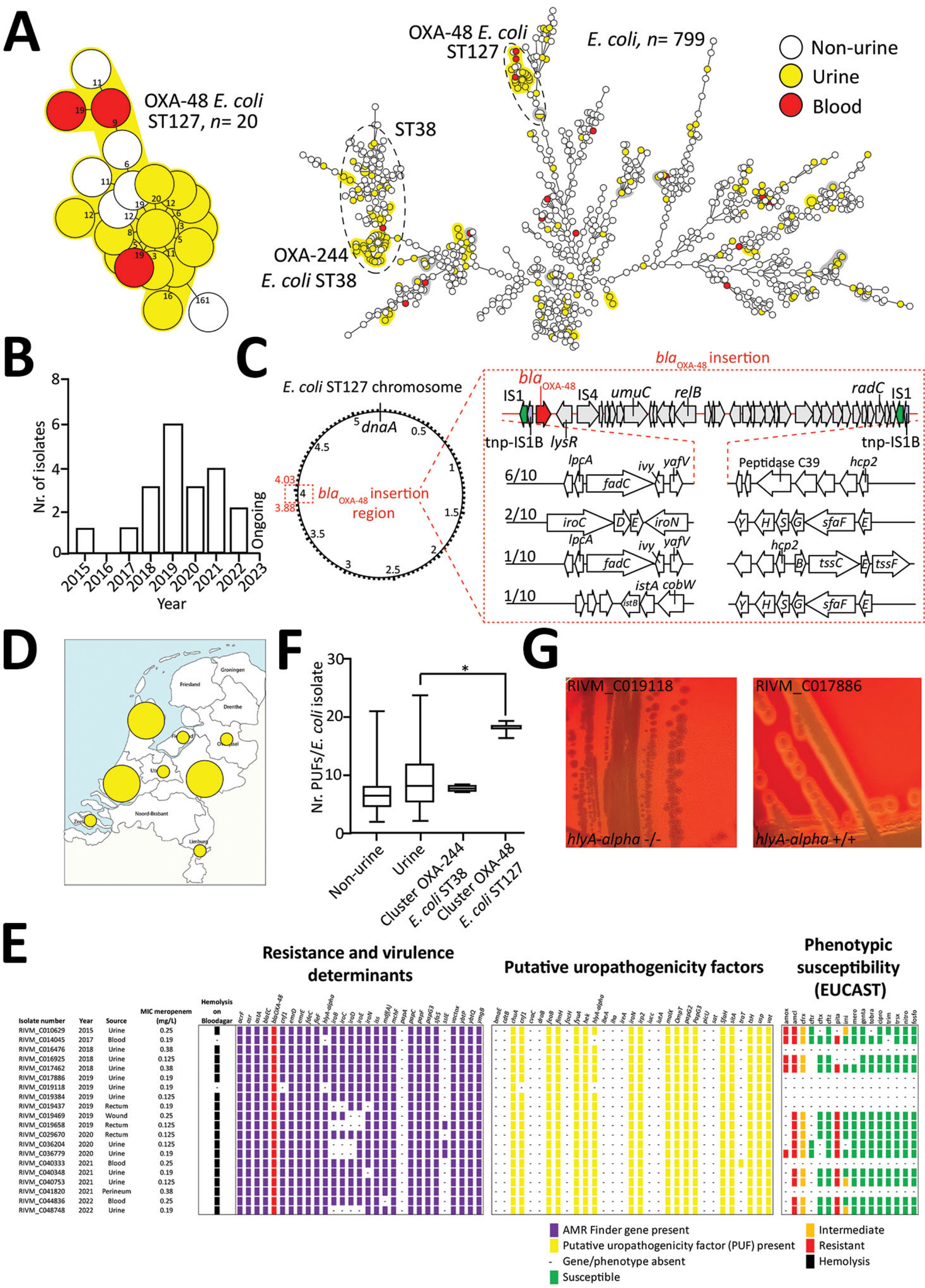

Appendix 1 Figure. Dissemination of OXA-48-producing UPEC ST127 in the Netherlands. A) Whole-genome multilocus sequence typing (wgMLST)–based minimum-spanning tree (MST) of 799 carbapenemase-producing *Escherichia coli* isolates, showing a genetic cluster (>2 isolates varying by <25 wgMLST alleles) of OXA-48 producing uropathogenic *E. coli* sequence type (ST) 127 in relation to other sequenced *E. coli* isolates from the Dutch CPE surveillance among isolates from urine, blood, and

previously reported OXA–producing *E.coli* ST38 cluster (*1*). Genetic relationship between the isolates is indicated by wgMLST allelic differences, and each circle represents an isolate. Yellow, isolated from urine; red, isolated from blood. The MST was based on an in-house *E. coli* wgMLST scheme described previously (*2*). B) Number of isolates of this cluster, which were sent to the National Institute for Public Health and the Environment (RIVM) per year. C) *E. coli* ST127 chromosome and variable chromosomal region of ≈148-kb indicating *bla*OXA-48 insertion, depicted in red. Numbers on the chromosome are in Megabase units, numbers in the red insert indicate number of chromosomes out of 10 complete assemblies with specific genetic make-up. Gene names are indicated. D) Geographic distribution of the isolates among 8/12 provinces.in the Netherlands. E) Characteristics of *E. coli* ST127 cluster isolates. Putative uropathogenicity factors (PUFs), genes according to AMRfinder, and phenotypic susceptibility (as tested according to the European Committee on Antimicrobial Susceptibility Testing) of the isolates. F) number of PUFs in comparison with other *E. coli* isolates from the Netherlands (nonurine, urine, and previous reported OXA-244 producing *E. coli* ST38 cluster). G) Representative image of β-hemolysis on tryptic soy blood agar when the *hlyA*-α gene in *E. coli* ST127 is present (right panel) and when the *hlyA*-α gene is absent (left panel).
